# 2,2-Dibromo-*N*-(4-fluoro­phen­yl)acetamide

**DOI:** 10.1107/S1600536812021174

**Published:** 2012-05-19

**Authors:** Xiangjun Qian, Zheng Fang, Shuxin Bao, Kai Guo, Ping Wei

**Affiliations:** a School of Pharmaceutical Sciences, Nanjing University of Technology, Xinmofan Road No. 5 Nanjing, Nanjing 210009, People’s Republic of China; b College of Life Science and Pharmaceutical Engineering, Nanjing University of Technology, Xinmofan Road No. 5 Nanjing, Nanjing 210009, People’s Republic of China

## Abstract

In the crystal structure of the title compound, C_8_H_6_Br_2_FNO, C—H⋯O and N—H⋯O hydrogen bonding results in six-membered rings and links the mol­ecules into chains running parallel to the *c* axis. The dihedral angle between the fluoro­phenyl ring and the acetamide group is 29.5 (5)°.

## Related literature

For background information, see: Feng *et al.* (2012 ▶). For related crystal structures, see: Gowda *et al.* (2009 ▶); Feng *et al.* (2012 ▶).
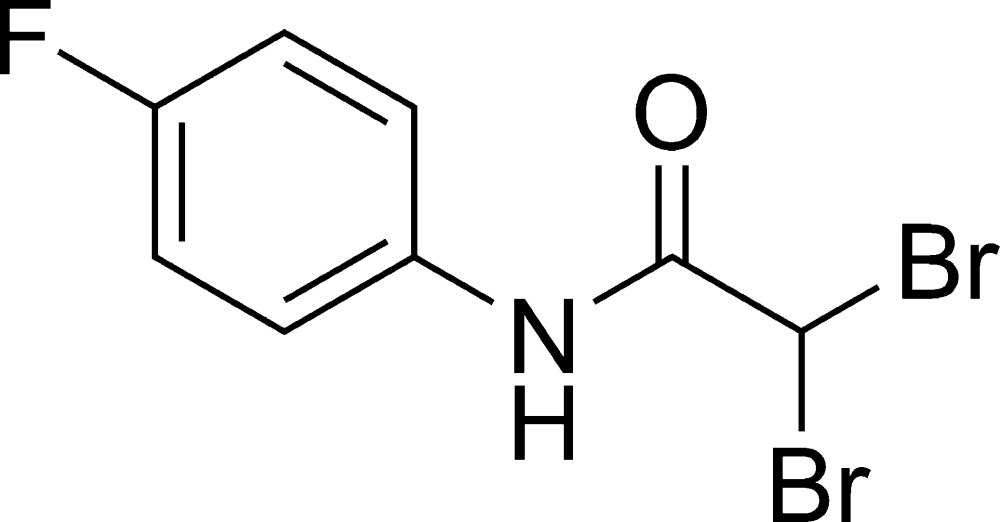


## Experimental

### 

#### Crystal data

C_8_H_6_Br_2_FNO
*M* *_r_* = 310.96
Monoclinic, 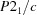

*a* = 9.746 (2) Å
*b* = 10.980 (2) Å
*c* = 9.426 (2) Å
β = 96.33 (3)°
*V* = 1002.5 (3) Å^3^
*Z* = 4
Mo *K*α radiation
μ = 8.06 mm^−1^
*T* = 293 K
0.10 × 0.10 × 0.10 mm

#### Data collection

Enraf–Nonious CAD-4 diffractometer
Absorption correction: ψ scan (North *et al.*, 1968 ▶) *T*_min_ = 0.975, *T*_max_ = 0.991
1937 measured reflections
1827 independent reflections
900 reflections with *I* > 2σ(*I*)
*R*_int_ = 0.068
3 standard reflections every 200 reflections intensity decay: 1%

#### Refinement

*R*[*F* ^2^ > 2σ(*F* ^2^)] = 0.058
*wR*(*F* ^2^) = 0.094
*S* = 1.00
1827 reflections
118 parameters
H-atom parameters constrained
Δρ_max_ = 0.49 e Å^−3^
Δρ_min_ = −0.50 e Å^−3^

### 

Data collection: *CAD-4 Software* (Enraf–Nonius, 1989 ▶); cell refinement: *CAD-4 Software*; data reduction: *XCAD4* (Harms & Wocadlo, 1995 ▶); program(s) used to solve structure: *SHELXS97* (Sheldrick, 2008 ▶); program(s) used to refine structure: *SHELXL97* (Sheldrick, 2008 ▶); molecular graphics: *SHELXTL* (Sheldrick, 2008 ▶) and *ORTEP-3* (Farrugia, 1997 ▶); software used to prepare material for publication: *PLATON* (Spek, 2009 ▶).

## Supplementary Material

Crystal structure: contains datablock(s) global, I. DOI: 10.1107/S1600536812021174/pv2533sup1.cif

Structure factors: contains datablock(s) I. DOI: 10.1107/S1600536812021174/pv2533Isup2.hkl

Additional supplementary materials:  crystallographic information; 3D view; checkCIF report

## Figures and Tables

**Table 1:** Hydrogen-bond geometry (Å, °)

*D*—H⋯*A*	*D*—H	H⋯*A*	*D*⋯*A*	*D*—H⋯*A*
N—H0*A*⋯O^i^	0.86	2.06	2.868 (7)	156
C1—H1*A*⋯O^i^	0.98	2.37	3.178 (9)	140

Symmetry code: (i) 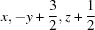.

**Fig. 1.**
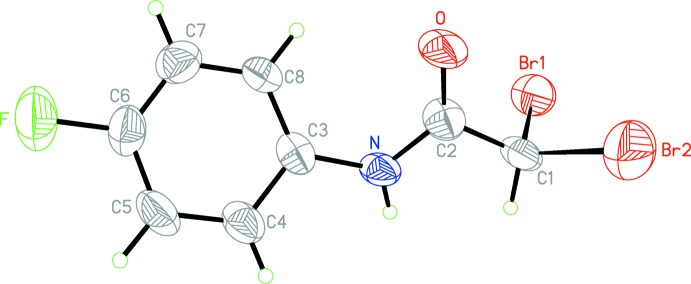
The molecular structure of the title compound with the atom numbering scheme. Displacement ellipsoids are drawn at the 30% probability level. H atoms are presented as small spheres of arbitrary radius.

**Fig. 2.**
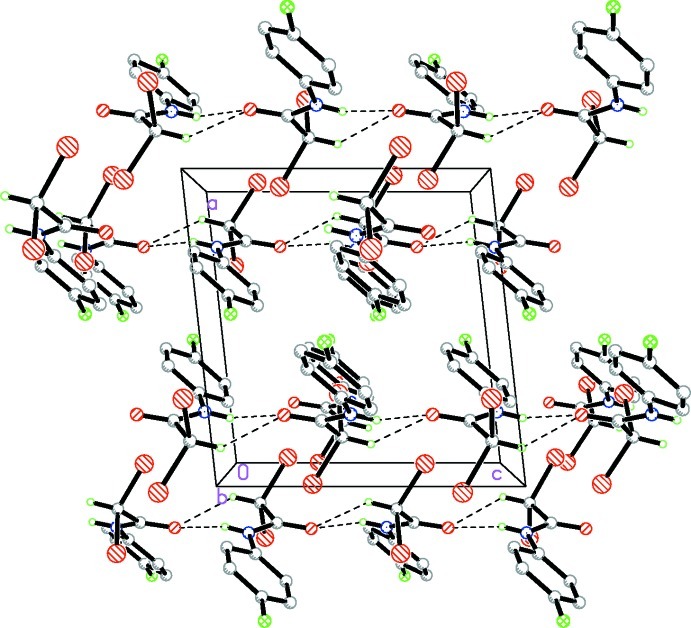
A view of the N—H···O and C—H···Ohydrogen bonds (dotted lines) in the crystal structure of the title compound.

## supplementary crystallographic information

### Comment

As a part of our studies on the synthesis of Ezetimibe (Fang *et al.*, 2012), the title compound which is one of the derivates of an intermediate, has been synthesized and its crystal structure is reported in this paper.

In the title molecule (Fig. 1), the dihedral angle between fluorophenyl ring (F/C3–C8) and acetamide group (O/N/C1/C2) group is 29.5 (5)°. The carbonyl O atom is hydrogen bonded to hydrogen atoms at N and C1, resulting in six membered rings linking the molecules into chains running parallel to the *c*-axis (Fig. 2 and Tab. 1). The bond distances and angles in the title molecule are in excellent agreement with the corresponding bond distances and angles reported in closely related structures (Gowda *et al.*, 2009; Feng *et al.*, 2012).

### Experimental

To 3-ethoxy-*N*-(4-fluorophenyl)acrylamide (1 g) was added 1,4-dioxane (20 ml) and water (20 ml) in a 50 ml flask. The solution was cooled to 273 K in an ice bath and *N*-bromosuccinimide (1.6 g) was added after 30 minutes. The solution was stirred at room temperature for 3 h. Then, the solution was heated to 353 K, after 40 minutes, the resulting mixture was concentrated under vacuum, the solid was collected by vacuum filtration, washed with cold water. Finally, the product was separated by silica gel column (yield = 59%). Crystals of the title compound suitable for X-ray diffraction were obtained by slow evaporation of an ethanol solution.

### Refinement

All H atoms were positioned geometrically and refined using a riding model, with N—H = 0.86 Å and C—H = 0.93 and 0.98 Å, for aryl and methyne H-atoms, respectively. The *U*_iso_(H) were allowed at 1.2*U*_eq_(N/C).

### Crystal data

**Table:**

C_8_H_6_Br_2_FNO	*F*(000) = 592
*M**_r_* = 310.96	*D*_x_ = 2.060 Mg m^−^^3^
Monoclinic, *P*2_1_/*c*	Mo *K*α radiation, λ = 0.71073 Å
Hall symbol: -P 2ybc	Cell parameters from 25 reflections
*a* = 9.746 (2) Å	θ = 9–12°
*b* = 10.980 (2) Å	µ = 8.06 mm^−^^1^
*c* = 9.426 (2) Å	*T* = 293 K
β = 96.33 (3)°	Block, colorless
*V* = 1002.5 (3) Å^3^	0.10 × 0.10 × 0.10 mm
*Z* = 4	

### Data collection

**Table:**

Enraf–Nonious CAD-4 diffractometer	900 reflections with *I* > 2σ(*I*)
Radiation source: fine-focus sealed tube	*R*_int_ = 0.068
Graphite monochromator	θ_max_ = 25.3°, θ_min_ = 2.1°
ω/2θ scans	*h* = −11→0
Absorption correction: ψ scan (North *et al.*, 1968)	*k* = 0→13
*T*_min_ = 0.975, *T*_max_ = 0.991	*l* = −11→11
1937 measured reflections	3 standard reflections every 200 reflections
1827 independent reflections	intensity decay: 1%

### Refinement

**Table:**

Refinement on *F*^2^	Primary atom site location: structure-invariant direct methods
Least-squares matrix: full	Secondary atom site location: difference Fourier map
*R*[*F*^2^ > 2σ(*F*^2^)] = 0.058	Hydrogen site location: inferred from neighbouring sites
*wR*(*F*^2^) = 0.094	H-atom parameters constrained
*S* = 1.00	*w* = 1/[σ^2^(*F*_o_^2^) + (0.024*P*)^2^] where *P* = (*F*_o_^2^ + 2*F*_c_^2^)/3
1827 reflections	(Δ/σ)_max_ < 0.001
118 parameters	Δρ_max_ = 0.49 e Å^−^^3^
0 restraints	Δρ_min_ = −0.50 e Å^−^^3^

### Special details

**Table:**

Geometry. All e.s.d.'s (except the e.s.d. in the dihedral angle between two l.s. planes) are estimated using the full covariance matrix. The cell e.s.d.'s are taken into account individually in the estimation of e.s.d.'s in distances, angles and torsion angles; correlations between e.s.d.'s in cell parameters are only used when they are defined by crystal symmetry. An approximate (isotropic) treatment of cell e.s.d.'s is used for estimating e.s.d.'s involving l.s. planes.
Refinement. Refinement of *F*^2^ against ALL reflections. The weighted *R*-factor *wR* and goodness of fit *S* are based on *F*^2^, conventional *R*-factors *R* are based on *F*, with *F* set to zero for negative *F*^2^. The threshold expression of *F*^2^ > σ(*F*^2^) is used only for calculating *R*-factors(gt) *etc*. and is not relevant to the choice of reflections for refinement. *R*-factors based on *F*^2^ are statistically about twice as large as those based on *F*, and *R*- factors based on ALL data will be even larger.

### Fractional atomic coordinates and isotropic or equivalent isotropic displacement parameters (Å^2^)

**Table:**

	*x*	*y*	*z*	*U*_iso_*/*U*_eq_	
F	0.4558 (5)	0.2056 (4)	0.4060 (6)	0.0868 (17)	
Br1	0.27410 (10)	0.98435 (8)	0.42192 (10)	0.0675 (3)	
Br2	−0.02715 (10)	0.90635 (10)	0.29891 (11)	0.0840 (4)	
O	0.2133 (6)	0.7340 (5)	0.2350 (5)	0.0635 (17)	
N	0.2193 (6)	0.6579 (5)	0.4588 (6)	0.0439 (17)	
H0A	0.1995	0.6743	0.5434	0.053*	
C1	0.1342 (8)	0.8605 (6)	0.4178 (8)	0.048 (2)	
H1A	0.1111	0.8455	0.5149	0.058*	
C2	0.1936 (8)	0.7438 (7)	0.3585 (8)	0.046 (2)	
C3	0.2775 (8)	0.5405 (6)	0.4353 (8)	0.0390 (19)	
C4	0.2452 (8)	0.4467 (7)	0.5254 (8)	0.052 (2)	
H4A	0.1850	0.4610	0.5936	0.062*	
C5	0.3007 (9)	0.3355 (8)	0.5140 (9)	0.059 (3)	
H5A	0.2770	0.2713	0.5708	0.070*	
C6	0.3943 (8)	0.3195 (8)	0.4150 (10)	0.054 (2)	
C7	0.4260 (8)	0.4084 (8)	0.3246 (8)	0.056 (2)	
H7A	0.4856	0.3933	0.2561	0.067*	
C8	0.3694 (8)	0.5186 (7)	0.3364 (7)	0.046 (2)	
H8A	0.3923	0.5815	0.2771	0.055*	

### Atomic displacement parameters (Å^2^)

**Table:**

	*U*^11^	*U*^22^	*U*^33^	*U*^12^	*U*^13^	*U*^23^
F	0.081 (4)	0.047 (3)	0.135 (5)	0.008 (3)	0.025 (3)	−0.004 (3)
Br1	0.0902 (7)	0.0504 (6)	0.0640 (6)	−0.0033 (6)	0.0179 (5)	−0.0068 (5)
Br2	0.0681 (7)	0.1079 (10)	0.0749 (8)	0.0201 (7)	0.0034 (5)	0.0095 (7)
O	0.116 (5)	0.052 (4)	0.026 (3)	0.013 (4)	0.020 (3)	−0.002 (3)
N	0.067 (5)	0.037 (4)	0.030 (4)	−0.005 (4)	0.016 (3)	−0.003 (3)
C1	0.075 (6)	0.037 (5)	0.035 (5)	−0.001 (4)	0.017 (4)	0.007 (4)
C2	0.063 (6)	0.046 (5)	0.029 (5)	0.001 (5)	−0.002 (4)	−0.007 (5)
C3	0.050 (5)	0.036 (5)	0.028 (5)	−0.006 (4)	−0.010 (4)	−0.001 (4)
C4	0.079 (7)	0.042 (6)	0.036 (5)	−0.004 (5)	0.009 (5)	0.004 (4)
C5	0.063 (6)	0.039 (6)	0.075 (7)	−0.014 (5)	0.012 (5)	0.014 (5)
C6	0.043 (5)	0.040 (6)	0.078 (7)	0.004 (5)	0.007 (5)	−0.004 (5)
C7	0.064 (6)	0.054 (6)	0.051 (6)	−0.005 (5)	0.014 (5)	−0.011 (5)
C8	0.056 (5)	0.044 (5)	0.037 (5)	0.000 (5)	0.009 (4)	0.010 (4)

### Geometric parameters (Å, º)

**Table:**

F—C6	1.394 (8)	C3—C4	1.392 (9)
Br1—C1	1.923 (7)	C4—C5	1.344 (9)
Br2—C1	1.896 (7)	C4—H4A	0.9300
O—C2	1.205 (7)	C5—C6	1.386 (10)
N—C2	1.339 (8)	C5—H5A	0.9300
N—C3	1.436 (8)	C6—C7	1.354 (10)
N—H0A	0.8600	C7—C8	1.340 (9)
C1—C2	1.536 (10)	C7—H7A	0.9300
C1—H1A	0.9800	C8—H8A	0.9300
C3—C8	1.384 (9)		
			
C2—N—C3	124.8 (6)	C5—C4—C3	120.2 (8)
C2—N—H0A	117.6	C5—C4—H4A	119.9
C3—N—H0A	117.6	C3—C4—H4A	119.9
C2—C1—Br2	109.1 (5)	C4—C5—C6	118.0 (8)
C2—C1—Br1	107.6 (5)	C4—C5—H5A	121.0
Br2—C1—Br1	111.3 (3)	C6—C5—H5A	121.0
C2—C1—H1A	109.6	C7—C6—C5	123.1 (8)
Br2—C1—H1A	109.6	C7—C6—F	118.6 (8)
Br1—C1—H1A	109.6	C5—C6—F	118.3 (8)
O—C2—N	125.6 (8)	C8—C7—C6	118.3 (8)
O—C2—C1	122.2 (7)	C8—C7—H7A	120.8
N—C2—C1	112.3 (6)	C6—C7—H7A	120.8
C8—C3—C4	119.3 (7)	C7—C8—C3	121.0 (7)
C8—C3—N	123.7 (7)	C7—C8—H8A	119.5
C4—C3—N	116.8 (7)	C3—C8—H8A	119.5
			
C3—N—C2—O	1.4 (13)	N—C3—C4—C5	−177.0 (7)
C3—N—C2—C1	−178.8 (6)	C3—C4—C5—C6	2.7 (13)
Br2—C1—C2—O	50.2 (9)	C4—C5—C6—C7	−3.8 (14)
Br1—C1—C2—O	−70.7 (9)	C4—C5—C6—F	177.9 (7)
Br2—C1—C2—N	−129.7 (6)	C5—C6—C7—C8	3.4 (13)
Br1—C1—C2—N	109.4 (6)	F—C6—C7—C8	−178.3 (7)
C2—N—C3—C8	30.9 (11)	C6—C7—C8—C3	−2.0 (12)
C2—N—C3—C4	−153.6 (7)	C4—C3—C8—C7	1.0 (11)
C8—C3—C4—C5	−1.4 (12)	N—C3—C8—C7	176.4 (7)

### Hydrogen-bond geometry (Å, º)

**Table:**

*D*—H···*A*	*D*—H	H···*A*	*D*···*A*	*D*—H···*A*
N—H0*A*···O^i^	0.86	2.06	2.868 (7)	156
C1—H1*A*···O^i^	0.98	2.37	3.178 (9)	140
C8—H8*A*···O	0.93	2.42	2.916 (9)	113

Symmetry code: (i) *x*, −*y*+3/2, *z*+1/2.
